# Somatic Donor Cell Type Correlates with Embryonic, but Not Extra-Embryonic, Gene Expression in Postimplantation Cloned Embryos

**DOI:** 10.1371/journal.pone.0076422

**Published:** 2013-10-16

**Authors:** Ryutaro Hirasawa, Shogo Matoba, Kimiko Inoue, Atsuo Ogura

**Affiliations:** 1 RIKEN BioResouce Center, Tsukuba, Ibaraki, Japan; 2 Graduate School of Life and Environmental Science, University of Tsukuba, Tsukuba, Ibaraki, Japan; 3 Center for Disease Biology and Integrative Medicine, Faculty of Medicine, University of Tokyo, Bunkyo-ku, Tokyo, Japan; Wellcome Trust Centre for Stem Cell Research, United Kingdom

## Abstract

The great majority of embryos generated by somatic cell nuclear transfer (SCNT) display defined abnormal phenotypes after implantation, such as an increased likelihood of death and abnormal placentation. To gain better insight into the underlying mechanisms, we analyzed genome-wide gene expression profiles of day 6.5 postimplantation mouse embryos cloned from three different cell types (cumulus cells, neonatal Sertoli cells and fibroblasts). The embryos retrieved from the uteri were separated into embryonic (epiblast) and extraembryonic (extraembryonic ectoderm and ectoplacental cone) tissues and were subjected to gene microarray analysis. Genotype- and sex-matched embryos produced by *in vitro* fertilization were used as controls. Principal component analysis revealed that whereas the gene expression patterns in the embryonic tissues varied according to the donor cell type, those in extraembryonic tissues were relatively consistent across all groups. Within each group, the embryonic tissues had more differentially expressed genes (DEGs) (>2-fold vs. controls) than did the extraembryonic tissues (*P*<1.0×10^–26^). In the embryonic tissues, one of the common abnormalities was upregulation of *Dlk1*, a paternally imprinted gene. This might be a potential cause of the occasional placenta-only conceptuses seen in SCNT-generated mouse embryos (1–5% per embryos transferred in our laboratory), because dysregulation of the same gene is known to cause developmental failure of embryos derived from induced pluripotent stem cells. There were also some DEGs in the extraembryonic tissues, which might explain the poor development of SCNT-derived placentas at early stages. These findings suggest that SCNT affects the embryonic and extraembryonic development differentially and might cause further deterioration in the embryonic lineage in a donor cell-specific manner. This could explain donor cell-dependent variations in cloning efficiency using SCNT.

## Introduction

Somatic cell nuclear transfer (SCNT) is a unique technology that produces a cloned animal from a single donor somatic cell nucleus. This technology is potentially promising for many practical applications in the fields of medicine and drug manufacturing, the animal industries and in conservation of genetic resources [1]. To achieve these goals, the somatic cell genomes should be precisely reprogrammed into the state seen in normally fertilized embryos: so-called totipotency. This prerequisite is quite different from that of other types of genomic reprogramming that give rise to a pluripotent state such as induced pluripotent stem (iPS) cell derivation or cell hybrid systems [2], [3]. Although pluripotent stem cells are undifferentiated cells that have the ability to differentiate into derivatives of all three primordial germ layers, they are somatic cells in a broad sense because they share some important characteristics in common with other somatic cells [4]. Conversely, for the achievement of totipotency the donor genome should be advanced beyond the epigenetic barriers that exist between the genomic states of the pre- and postimplantation embryo stages. This epigenetic barrier might comprise somatic cell type ‘memories’ such as large-scale or region-specific DNA methylation and repressive histone methylation, all of which are maintained in the somatic cell genome throughout life [4]. During the natural course of development, these somatic memories are erased or reprogrammed in primordial germ cells shortly after germline specification [5]. Therefore, most – if not all – of them cannot be reprogrammed sufficiently within the cytoplasm of mature oocytes in the course of conventional SCNT procedures. This probably constitutes one of the major obstacles associated with mammalian SCNT. Indeed, it has been reported that preimplantation cloned SCNT-derived embryos are prone to several epigenetic abnormalities such as global DNA hypermethylation [6], satellite repeat DNA hypermethylation [7], [8] and histone methylation [9], which are thought to be legacies from the somatic cell genome.

The presence of such epigenetic abnormalities related to insufficient or erroneous reprogramming in SCNT-derived embryos has prompted researchers to investigate their gene expression profiles in depth. For bovine and mouse embryos, microarray technology in conjunction with linear amplification of mRNA from individual embryos has been employed extensively to clarify the gene expression patterns specific to SCNT [10]–[13]. Thus, the gene expression patterns of bovine SCNT-derived blastocysts closely resembled those of naturally fertilized embryos, indicating that the bovine clones underwent genomic reprogramming efficiently [11]. By contrast, SCNT-derived mouse blastocysts revealed a number of dysregulated genes when compared with genotype- and sex-matched control embryos. Interestingly, two independent groups reported an overlapping set of commonly dysregulated genes across different donor cell types, especially on the X chromosome [12], [13]. Furthermore, gene knockout experiments confirmed that most (about 80–85%) of the downregulated genes could be attributable to ectopic expression of *Xist*, a gene responsible for X chromosome inactivation [12]. Another defined defect found specifically in mouse SCNT-derived blastocysts was a remarkable repression of the *Magea* and *Xlr* gene clusters, localized in chromosomal regions XqF3 and XqA7.2–7.3, respectively [12]. These regions are within genomic blocks enriched with dimethylation of histone H3 at lysine 9 (H3K9me2) [14], an epigenetic state that is responsible for gene silencing and that establishes a local heterochromatin domain.

Despite the abnormal epigenetic and gene expression profiles described above, many mouse SCNT-derived embryos reach the blastocyst stage and subsequently undergo implantation [15]–[17], as shown by a decidualized endometrium with occasional remnants of invading blastocysts [18]. However, immediately after implantation most start to show developmental arrest and a series of SCNT-specific abnormal phenotypes [16]–[18]. Our knockdown experiments using *Xist*-specific short interfering (si) RNA indicated that many of the developmental failures during the early postimplantation period could be rescued by correction of aberrant *Xist* expression before implantation [19]. However, such siRNA-treated embryos and even *Xist* knockout embryos still show higher incidences of developmental failure and of SCNT-specific abnormal phenotypes such as placental development lacking a fetus and placental hyperplasia (unpublished data). Therefore, there must be some *Xist*-independent mechanisms that affect the postimplantation development of SCNT-derived embryos. To gain better insight into this etiology, here we analyzed the genome-wide gene expression profiles of embryonic day (E)6.5 mouse postimplantation embryos cloned from three different cell types (cumulus cells, neonatal Sertoli cells and fibroblasts). As we inferred in our previous papers, ectopic *Xist* expression in SCNT-derived embryos is a consequence of the discrepancy of *Xist* regulation between somatic cells and early embryos, but is not a result of abnormal genomic reprogramming. Therefore, we expected that by using *Xist* gene knockout we could narrow the gene expression profiles that might more exactly reflect the cell type-specific reprogramming pattern at the time of SCNT. Therefore, in this study we generated cloned embryos of both sexes from *Xist* knockout donor cells. Embryos retrieved from the uteri of recipient foster mothers were separated into the embryonic (epiblast) and extraembryonic (extraembryonic ectoderm and ectoplacental cone) tissues and the data were compared with those from genotype- and sex-matched control embryos produced by *in vitro* fertilization (IVF).

## Materials and Methods

### Animals

C57BL/6N (B6), DBA/2 and (C57BL/6N × DBA/2)F1 (BDF1) strains of mice were purchased from Japan SLC, Inc. (Shizuoka, Japan) and ICR mice were purchased from CLEA Japan, Inc. (Tokyo, Japan). For somatic cell nuclear donors, heterozygous *Xist* knockout mice [20] from a BDF1 background were produced by crossing heterozygous *Xist* knockout B6 female mice with DBA/2 male mice [12]. All mice were maintained under specific-pathogen-free conditions and were housed under controlled lighting conditions (light: 0700–2100). All animal experiments described here were approved by the Animal Experimentation Committee at the RIKEN Tsukuba Institute and were performed in accordance with the committee's guiding principles.

### Collection of recipient oocytes

Eight- to 10-week-old BDF1 females were induced to superovulate by injections of 7.5 IU of pregnant mare serum gonadotropin (Sankyo, Tokyo, Japan) and 7.5 IU of human chorionic gonadotropin (hCG; Sankyo) at an interval of 48–50 h. Mature MII oocytes were collected from the oviducts 15 h after hCG injection and released from cumulus cells by treatment with 0.1% bovine testicular hyaluronidase (Merck Millipore Japan, Tokyo, Japan) in potassium simplex optimized medium (KSOM).

### Preparation of donor cells

All three types of nuclear donor cells (cumulus cells, neonatal Sertoli cells and fibroblasts) were prepared from heterozygous *Xist* knockout mice with a BDF1 genetic background (see above). Cumulus cells were collected from cumulus–oocyte complexes of superovulated adult female mice after treatment with KSOM containing 0.1% hyaluronidase. Neonatal Sertoli cells were prepared as a testicular suspension as described [19], [21]. In brief, testes of 1- to 9-day-old male mice were treated with 0.1 mg/ml collagenase (Sigma-Aldrich, St. Louis, MO, USA) for 30 min at 37°C followed by 0.2 mg/ml trypsin (Sigma-Aldrich) for 5 min at 37°C. The dissociated testicular cells were washed and suspended with phosphate-buffered saline (PBS) containing 4 mg/ml bovine serum albumin (Merck Millipore Japan) and used for injection. Fibroblasts were prepared from adult female tail-tip tissues as described [22]. Pieces of tail-tip tissue were placed on the bottom of culture dishes and cultured in Dulbecco's modified Eagle's medium (DMEM) containing 10% fetal bovine serum (FBS) at 37°C in a humidified atmosphere of 5% CO_2_ in air for 1–2 weeks until the fibroblasts expanded and became confluent.

### SCNT

Nuclear transfer from cumulus or neonatal Sertoli cells was carried out using a Piezo-driven micromanipulator (PMM-150FU, Prime Tech Ltd., Ibaraki, Japan) as described [21], [23]. Briefly, recipient oocytes collected from BDF1 female mice were enucleated in Hepes-buffered KSOM containing 7.5 µg/ml cytochalasin B (Merck Millipore Japan). Thereafter, the donor cumulus or Sertoli cell nuclei were injected into enucleated oocytes. Fibroblast nuclear transfer was performed by electrofusion of donor cells with enucleated oocytes [22]. A single fibroblast was inserted into the perivitelline space of the enucleated oocyte. Fusion was induced by a DC pulse of 1800 V/cm for 10 microseconds with pre- and postpulse AC (300 V/cm, 2 MHz, 30 seconds each) using ECM200 (BTX, San Diego, CA, USA) in 300 mM mannitol containing 0.1 mM MgSO_4_ and 0.1 mg/ml polyvinyl alcohol. After 1 h culture in KSOM, the reconstructed oocytes were activated with 3 mM SrCl_2_ for 1 h and cultured in KSOM containing 5 µg/ml cytochalasin B for 5 h. They were further cultured in KSOM for 12–18 h until they reached the 2-cell stage. BDF1 embryos generated by conventional IVF were used as controls.

To determine whether the method of nuclear transfer (microinjection or membrane fusion) could cause differences in the transcriptomes of SCNT embryos, in a preliminary study we reconstructed embryos with cumulus cell nuclei either by injection or fusion and compared their gene expression profiles by microarray. As shown in Figure S1 in File S1, the global gene expression patterns of both SCNT groups were indistinguishable. Although we confirmed this only with cumulus cell-derived clones, this result indicates that the differences in transcriptomes among the donor cell type groups in this study most likely reflected the genomic reprogrammability specific for the donor cell type rather than technical factors during the SCNT procedure.

### Embryo transfer and recovery

Embryos that reached the 2-cell stage were transferred into the oviducts of ICR recipient mice at day 1 of pseudopregnancy (the day a copulatory plug was observed after mating with a vasectomized male mouse). On day 7 of pregnancy, corresponding to E6.5, the implanted embryos were recovered carefully from the uteri and isolated from decidua using fine forceps and a needle under a dissecting microscope. After incubation in PBS with 0.25% pancreatin and 0.05% trypsin for 10 minutes at 4°C, embryos were transferred to DMEM containing 10% FBS and then the visceral endoderm layer was detached from epiblast or extraembryonic ectoderm by being gently aspirated through a mouth pipette several times. Thereafter, the epiblast was separated from the extraembryonic ectoderm using a fine tungsten needle [24]. Isolated epiblasts or extraembryonic regions (extraembryonic ectoderm and ectoplacental cone) were subjected to microarray gene analyses.

### Gene microarray

Microarray analyses for gene expression were performed as described [12], [19]. Total RNA was extracted with TRIzol (Life Technologies Japan Ltd., Tokyo, Japan) from epiblasts or extraembryonic regions derived from single embryos and subjected to linear amplification using TargetAmp Two-Round Aminoallyl-aRNA Amplification Kits (Epicentre Biotechnologies, Madison, WI, USA). Amplified RNA was labeled with Cy3 dye (GE Healthcare UK Limited, Buckinghamshire, England) and hybridized to a whole mouse genome oligo DNA microarray (4×44 K, Agilent Technologies, Palo Alto, CA, USA) for 17–18 h at 65°C. The scanned images of microarray slides were processed using Feature Extraction software 10.5.1.1 (Agilent Technologies).

### Quantitative reverse transcription polymerase chain reaction (qPCR)

Total RNA was isolated using ISOGEN (Nippon Gene, Toyama, Japan) from embryonic and extraembryonic tissues of the E6.5 embryos. First-strand cDNA was synthesized using a SuperScript III First-Strand Synthesis System (Invitrogen, Life Technologies, Carlsbad, CA, USA) with random hexamer primers. All qPCR runs were performed using QuantiTect SYBR Green PCR kits (QIAGEN, Hilden, Germany) and ABI Prism 7900HT Sequence Detection System (Applied Biosystems, Inc., Foster City, CA, USA) with a condition of 40 cycles of 95°C for 20 s, 60°C for 20 s and 72°C for 20 s, following a denaturation step at 95°C for 10 min. Primers used are listed in Table S1.

### Statistical analysis

Statistical analyses of microarray data were performed using Gene Spring GX 12.5 software (Agilent Technologies). All raw data were subjected to quintile normalization. To reduce background noise, the genes of a microarray flagged with “not detected,” both in the SCNT and control samples, were removed from the data set. Consequently, about 30000 genes (probes) (27662 to 32001 out of 41267) were used for the statistical analysis (see Results). Normalized values were analyzed by moderated *t* tests with a 5% significance level and a 2.0-fold filter was applied to identify differentially expressed genes (DEGs) between SCNT-derived embryo samples and sex- and tissue-matched IVF-derived control samples. Microarray data were MIAME (Minimum Information About a Microarray Experiment) compliant and have been deposited in the GEO public database (GSE49173) (http://www.ncbi.nlm.nih.gov/geo/). Other statistical analyses are indicated in the text as appropriate.

## Results

### Validation of the mechanical isolation of embryonic and extraembryonic tissues by specific marker genes

**Figure 1:**
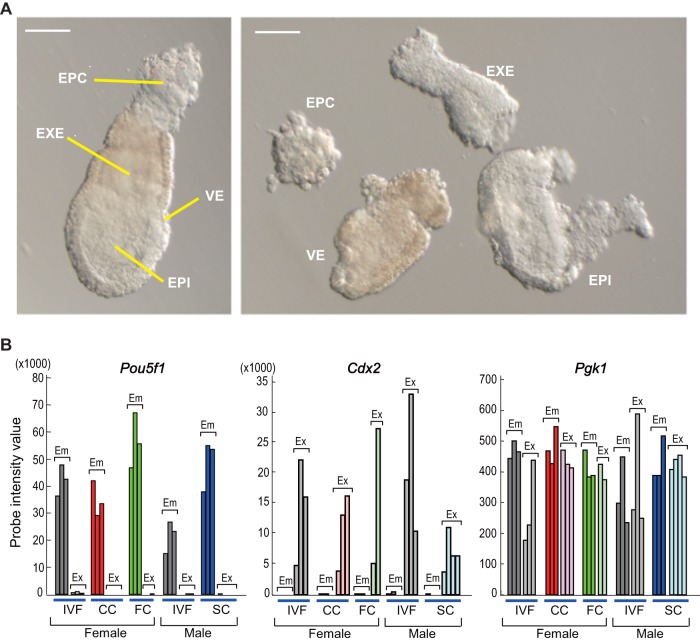
Preparation and validation of the samples. (**A**) Embryos were retrieved at E6.5 (left) and dissected into four parts (right). EPC, ectoplacental cone; VE, visceral endoderm; EXE, extraembryonic ectoderm; EPI, epiblast. Scale bar  = 100 µm. B) Raw signal values of *Pou5f1* (left), *Cdx2* (center) and *Pgk1* (right) genes extracted from microarray data. *Pou5f1* and *Cdx2* were detected exclusively in the embryonic and extraembryonic samples, respectively, while *Pgk1* was detected in both samples. These results confirmed the accuracy of sample preparation from the embryonic and extraembryonic tissues. Em, embryonic samples; Ex, extraembryonic samples; IVF, samples from *in vitro* fertilized control embryos; CC, cumulus cell-derived clone; FC, fibroblast-derived clone; SC, Sertoli cell-derived clone. See Figure S2 for further validation by several other marker genes.

The SCNT-derived embryos analyzed in this study are listed in Table 1. They were collected from the uteri at E6.5 (Figure 1A left) and were dissected into four parts: epiblast (EPI), extraembryonic (EXE), visceral endoderm (VE) and ectoplacental cone (EPC) (Figure 1A right). For subsequent analyses, the EPI part was treated as an embryonic sample and a mixture of EXE and EPC was treated as a pooled extraembryonic sample. The VE part was not used in this study. To validate the quality of the mRNA samples, we first checked the expression levels of markers for the embryonic and extraembryonic lineages. By comparing raw signal intensities, *Pou5f1* (also called *Oct4*), *Nanog*, *Fgf5*, *Nodal* and *Cripto* genes, which are expressed dominantly in the epiblast [25]–[27], showed high expression levels in the embryonic samples, while they were almost at the background level in the extraembryonic samples (Figures 1B and S2 in File S1). Conversely, *Cdx2*, *Elf5*, *Fgfr2*, *Plet1* and *Gjb5*, which are predominantly expressed in extraembryonic tissues [25], [28]–[29], showed a high level of expression in the extraembryonic samples, but not in the embryonic samples (Figures 1B and S2 in File S1). The ubiquitously expressed *Pgk1* gene was stably detected in both tissues (Figure 1B). These results confirm that there was no cross-contamination between the embryonic and extraembryonic samples. The results also suggest that these lineage marker genes might not be severely affected by the SCNT procedure, at least at the postimplantation stage.

**Table 1:** List of samples from E6.5 cloned embryos analyzed by microarray.

Donor cell type	Sex	Number of samples	
		Embryonic	Extraembryonic
Cumulus cells (CC)	Female	3	3
Tail-tip fibroblasts (FC)	Female	3	2
Neonatal Sertoli cells (SC)	Male	3	4
Control (IVF)	Female	3	3
Control (IVF)	Male	3	3

CC, cumulus cell-derived clone; FC, fibroblast-derived clone, SC: Sertoli cell-derived clone; IVF, *in vitro* fertilization-derived control embryos.

### Donor-specific gene expression pattern in the embryonic but not extraembryonic tissues

**Figure 2:**
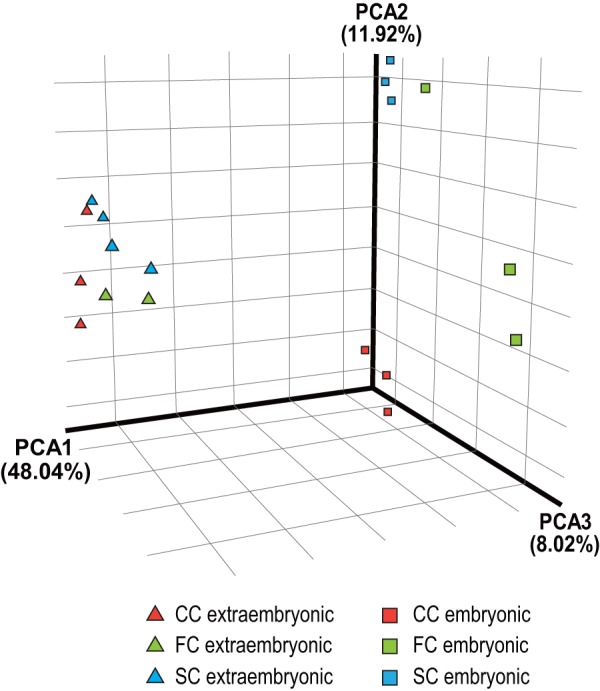
Principal component analysis (PCA) of global gene expression profiles. Visualization of gene expression similarities of samples clearly distinguishes donor cell-specific clusters of the embryonic samples (squares) from a donor cell-independent (i.e., SCNT-specific) overlapping cluster of extraembryonic samples (triangles). Red, green and blue represent cumulus cell-derived clone, fibroblast-derived clone and neonatal Sertoli cell-derived clone samples, respectively. Only the SCNT groups are presented based on the relative distance from the corresponding sex- and tissue type-matched IVF-derived control embryos. The result of conventional PCA, in which all IVF-derived samples were analyzed concomitantly, is shown in Figure S3.

We performed principal component analysis (PCA) to determine how the gene expression patterns were affected by the donor cell genome. For the embryonic tissues, the expression profiles could be clearly separated into three donor cell groups (squares in Figures 2 and S3 in File S1). This suggests that their impaired expression patterns represented epigenetic information derived from the donor cells, which remained after implantation. However, in extraembryonic tissues, the PCA plots were much closer to each other than those of embryonic samples, indicating much less variability in their expression profiles among the donor cell types (triangles in Figures 2 and S3 in File S1).

### Embryonic tissues showed more gene expression aberrations than extraembryonic tissues

**Figure 3:**
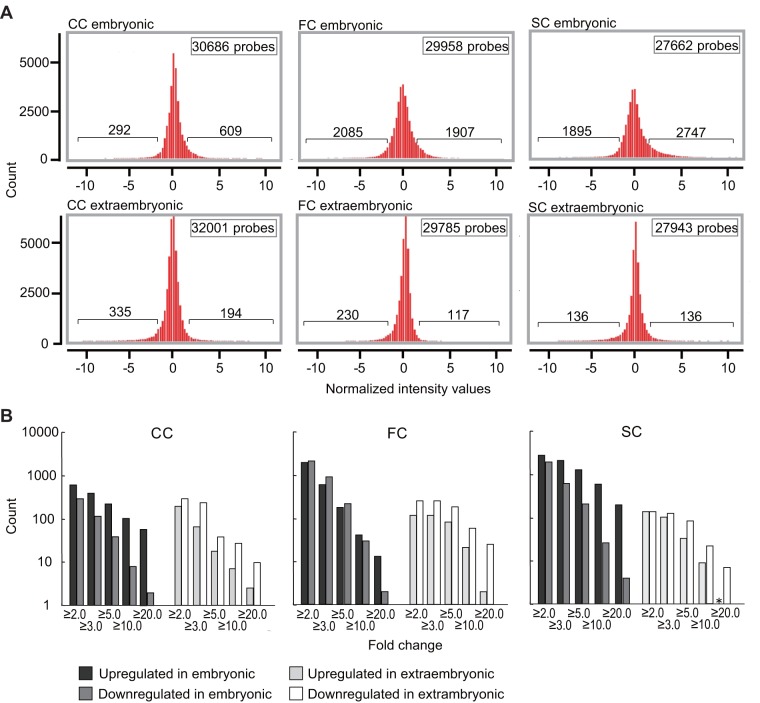
Fold change analysis of gene expression profiles of embryonic and extraembryonic samples. Fold change analysis with a cutoff of f >2 identified the number of differentially expressed genes (DEGs) in SCNT-derived samples compared with IVF-derived controls. (**A**) Histogram showing the distribution of DEGs classified by the relative values compared with the IVF-derived controls. The embryonic samples included more DEGs (901–4642) than the extraembryonic samples (272–592). This tendency is depicted by the narrower and higher distribution patterns of the extraembryonic samples. (**B**) The cumulative numbers of up- and downregulated DEGs shown side-by-side based on fold changes. Dark and light bars represent up- and downregulated DEGs, respectively. In the embryonic tissues, large fold changes occurred predominantly with the upregulated DEGs, whereas in the extraembryonic tissues such changes occurred with the downregulated DEGs. *No corresponding gene.

In the next analysis, we extracted DEGs between cloned and IVF-derived control embryos (cutoff: 2-fold). The extraembryonic samples contained fewer DEGs (272 to 529 genes, respectively) compared with embryonic samples (901–4642 genes) in all of the donor cell types (*P*<1.0×10^–26^ within the same donor cell group; Chi-squared test with Yates' correction; Figure 3A). This suggests that a large proportion of aberrant gene expression must be corrected precisely in extraembryonic tissues during or after implantation because our previous study showed that more than 1000 genes were different in blastocyst stage embryos [12]. We then looked at highly up- or downregulated genes side-by-side based on the fold change to evaluate quantitative changes in more detail (Figure 3B). In embryonic tissues, most of the DEGs were upregulated, whereas they were downregulated in extraembryonic tissues; moreover, this tendency was reinforced in genes with higher fold changes in all donor cell types. In other words, highly upregulated genes were more frequently found in embryonic tissues and highly downregulated genes were more frequently found in extraembryonic tissues. Taken together, these data suggest that gene expression dynamics were modulated actively in cloned extraembryonic tissues during and after implantation, although donor nuclear-derived aberration remained in the embryonic tissues.

### The expression levels of large organized chromatin K9 modification (LOCKs)-related genes were indistinguishable between IVF- and SCNT-derived embryos

**Figure 4:**
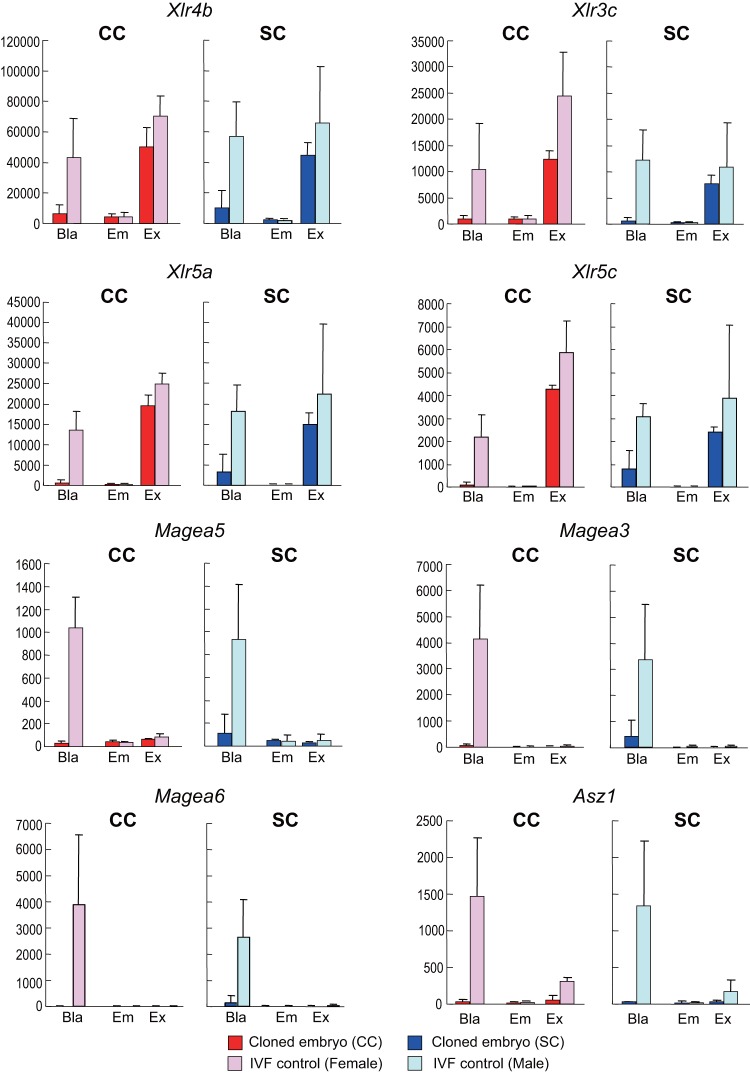
Expression of LOCKs-related genes in SCNT-derived embryos. Raw intensity values of LOCKs-related genes at E3.5 (blastocysts) and E6.5. The data set at E3.5 was from a previous study [12] (accession number: GSE23181). At E3.5, all the LOCKs genes examined were strongly repressed in SCNT-derived blastocysts compared with those in control IVF-derived blastocysts. At E6.5, after implantation, the genes were downregulated in the embryonic tissue and no significant differences were found between the SCNT- and IVF-derived embryos. By contrast, in the extraembryonic tissues, *Xlr* genes (e.g., *Xlr4b*, *Xlr3c*, *Xlr5a* and *Xlr5c*) remained active after implantation and in the SCNT-derived samples the expression levels were restored to nearly normal. Bla, blastocysts; Em, embryonic samples; Ex, extraembryonic samples; CC, cumulus cell-derived clone; SC, Sertoli cell-derived clone.

We next examined the expression patterns more specifically for some genes based on the results of microarray studies using SCNT-derived blastocysts. We and other groups have demonstrated previously that two discrete groups of genes are suppressed in mouse SCNT-derived blastocysts in a *Xist*-independent manner. These are the *Magea* and *Xlr* gene clusters, localized in chromosomal regions XqF3 and XqA7.2–7.3, respectively [12]. These regions are within the blocks called LOCKs, enriched with H3K9me2 [14]. The *Magea* and *Xlr* genes were actively transcribed in IVF-derived blastocysts, but became shut down in embryonic stem (ES) cells [12]. As expected, in the embryonic tissues from both IVF- and SCNT-derived samples, the *Magea* and *Xlr* genes were strongly repressed to the basal level (Figure 4). Expression of *Magea* genes (e.g., *Magea5*, *Magea3* and *Magea6*) was also repressed in the extraembryonic tissues, so there was no difference between the control and SCNT-derived embryos (Figure 4). By contrast, *Xlr* genes (e.g., *Xlr4b*, *Xlr3c*, *Xlr5a* and *Xlr5c*) were expressed in both IVF- and SCNT-derived embryos, indicating that the expression of *Xlr* had recovered in SCNT-derived embryos after implantation (Figure 4). *Asz1*, another LOCK gene on chromosome 6 [12], [13], showed patterns very similar to *Magea* (Figure 4).

### Consistent upregulation of *Dlk1* in the SCNT-derived embryonic tissues

**Figure 5:**
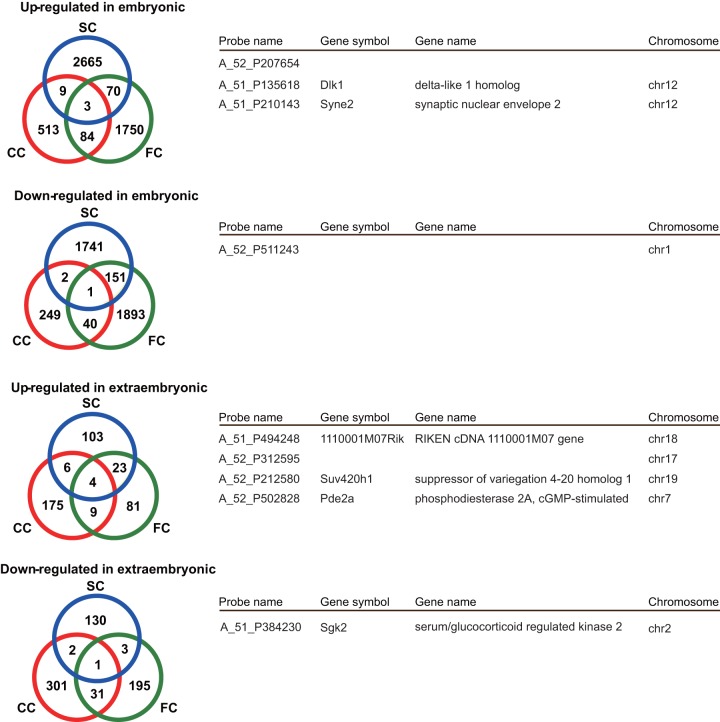
Overlapping DEGs among the three SCNT groups. (**A**) Venn diagrams of the DEGs identified in Figure 3 (fold change >2.0), showing the numbers of shared and unique DEGs among three different SCNT groups. There were very few DEGs shared with all three groups in both embryonic and extraembryonic tissues. (**B**) List of overlapping DEGs among the three SCNT groups. A complete list of DEGs is shown in Table S2.

To determine the key genes responsible for SCNT-specific abnormalities, we examined the overlap among three different SCNT donor cell groups. As shown in Figure 5, we found that there was minimal overlap between the groups. In the embryonic tissues, only three and one genes were shared by the upregulated and downregulated DEG sets, respectively (Figure 5A). Similarly, only four and one genes were shared by the upregulated and downregulated DEG sets, respectively, in the extraembryonic tissues (Figure 5B). We then examined whether these common DEGs had any functions in embryonic or extraembryonic development (listed in Figure 5). One of the most plausible genes in the abnormal development of SCNT-derived embryos is *Dlk1*, which was commonly upregulated in embryonic tissues. This is a paternally imprinted gene that plays an essential role in embryonic development [30]. The same expression pattern, namely a significant upregulation of *Dlk1* in the embryonic tissues and no significant dysregulation of other imprinted genes in the same cluster in either tissue, was also found by qPCR analysis (Figure S4 in File S1).

**Figure 6:**
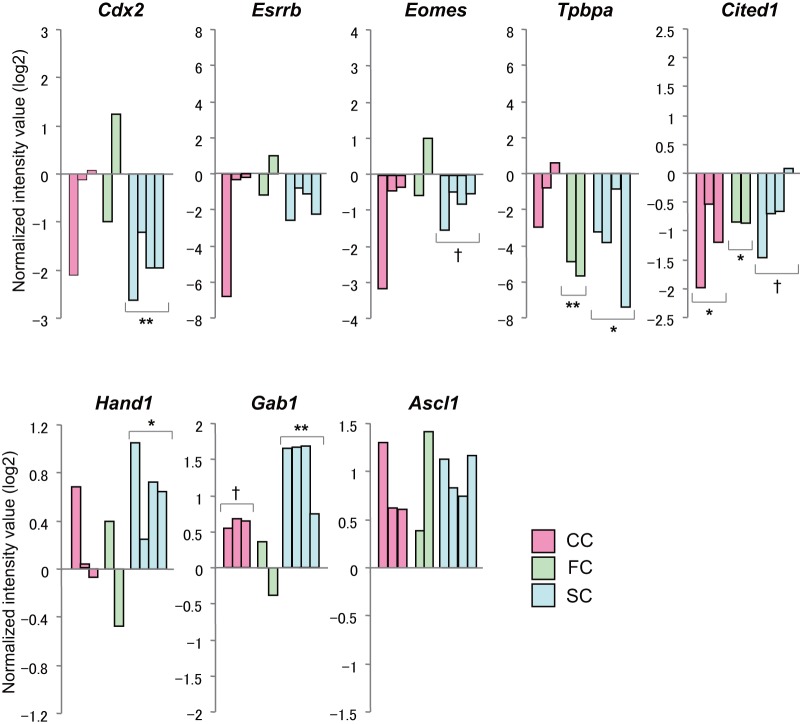
Expression levels of genes important for early placentation. Genes important for maintenance of undifferentiated trophoblast cells (*Cdx2*, *Esrrb* and *Eomes*) were downregulated while those essential for differentiation into giant cells (*Hand1*) were upregulated. * *P*<0.05, ** *P*<0.01 (compared with the corresponding IVF-derived controls). “†” indicates a tendency for down- or upregulation, but not with a statistical significance (*P*<0.10).

Among the five common DEGs in the extraembryonic tissue, there were no candidate genes that could account for the abnormal placental development in SCNT-derived embryos. We assumed that this might have arisen, at least in part, from unexpectedly high variations in the expression profiles in the control IVF-derived extraembryonic tissues. It is known that SCNT-derived placentas at early to midgestation stages are characterized by a decrease in undifferentiated diploid cells and an increase in trophoblastic giant cells [16], [18]. We then examined the expression levels of some genes essential for early placentation. As expected, *Cdx2*, *Esrrb*, and *Eomes*, which are expressed in undifferentiated extraembryonic tissues [31], [32], showed tendencies for decreased expression (Figure 6). *Tpbpa*, a marker for diploid trophoblast cells [31] and *Cited1*, a regulator of trophoblast layer formation [33], were also downregulated (Figure 6). By contrast, *Hand1*, which is known to be related to giant cell formation [34], seemed upregulated with a significant difference in the Sertoli cell group (Figure 6). We also noted that two imprinted genes, *Gab1* (unpublished data) and *Ascl1* (also called *Mash2*) [35], were upregulated (Figure 6). The complete list of DEGs with at least a 2-fold change in SCNT-derived embryos compared with control embryos is shown in Table S2.

## Discussion

It is very common that abnormal phenotypes appear predominantly after implantation following SCNT cloning in mammals. The present study was undertaken to determine whether there were any SCNT-specific aberrations in gene expression shortly after implantation that might explain such abnormal phenotypes. There have been many studies analyzing gene expression profiles in SCNT-derived embryos before implantation in different species, including mice, cattle and pigs [10]–[13], [36]. However, because of the complexity of the structure of postimplantation embryos and the technical difficulty in retrieving them intact from uteri, the precise gene expression profiles of SCNT-derived embryos shortly after implantation remain largely ill defined. In this study, for the first time we have performed DNA microarray analysis of early postimplantation SCNT-derived embryos, providing data on the embryonic and extraembryonic tissues separately. This success could be attributed to the technological precision and appropriate analytical approaches we employed. We mechanically separated the conceptuses into two parts: embryonic and extraembryonic tissues. This was confirmed by the discrete expression of genes specific to each component (Figures 1 and S2 in File S1). Besides this technical precision, another point that made this study biologically meaningful was the use of *Xist* knockout mice as nuclear donors. As we have shown previously, the *Xist* gene when expressed ectopically in mouse SCNT-derived embryos causes a global dysregulation of many development-related genes [12]. Using heterozygous *Xist* knockout mice as donors, the birth rates increased to about 10%, reflecting extensive correction of the global gene expression patterns [12]. The ectopic expression of *Xist* was probably caused by the lack of a *Xist*-repressing epigenetic imprint in the donor genome, but not by reprogramming errors within the recipient ooplasm [4]. Therefore, we expected that exclusion of the effect of excessive *Xist* from the microarray analysis would help towards better understanding of SCNT-associated abnormalities from the viewpoint of genomic reprogramming errors.

One of the interesting findings in this study was the differential responsiveness of embryonic and extraembryonic tissues to SCNT. As clearly demonstrated by PCA (Figure 2), genes in the embryonic tissues showed donor-specific expression patterns, whereas those in the extraembryonic tissues showed an SCNT-specific pattern independent of the donor cell type. This finding is consistent with previous histological observations of cloned conceptuses at E10.5 [18]. At this stage, when formation of the hemochorial placentation is complete, the cloned placentas displayed defined histological patterns reﬂecting their stages of developmental arrest. The most notable abnormality was the poor development of the spongiotrophoblast layer of diploid cells. By contrast, complex structural abnormalities are manifold in embryonic tissues at this time and all or at least some organs were affected by SCNT. It is known for mouse SCNT technology that cloning efficiency can vary with the donor cell type despite there being no correlation between the birth rate and the differentiation status of donor cells [4]. Therefore, it is possible that the gene expression pattern of the embryonic lineages, rather than that of the placental lineages, might be a better predictive parameter for the outcome of mouse SCNT cloning. This assumption is supported by another finding that the number of DEGs in the embryonic tissue was consistently greater than in the extraembryonic tissues in all three groups of clones analyzed. Taken together, we speculate that genomic reprogramming or reorganization of nuclear integrity during implantation – if any – might occur more extensively in the extraembryonic rather than the embryonic lineages. This could have resulted in mitigation of the donor-specific reprogramming errors in the placentas. Consistent with this hypothesis, the extraembryonic tissue of the postimplantation cloned embryos restored expression of the *Xlr* family genes, which are strongly repressed at the blastocyst stage in a *Xist*-independent manner [12], [13].

Here we sought to identify the genes responsible for placental abnormalities associated with mouse SCNT. It is known that cloned mouse placentas are characterized by hypoplasia of diploid trophoblastic cell layers in early stages and hyperplastic trophoblast cell proliferation in later stages [16], [18], [37], [38]. In this study, we obtained several informative results that could explain the poor development of SCNT-derived placental tissues at early postimplantation stages. Although not classified as DEGs common to all SCNT groups, genes important for the maintenance of undifferentiated trophoblast cells (*Cdx2*, *Esrrb* and *Eomes*) [31], [32] and diploid trophoblast cells (*Tpbpa*) were downregulated, while those essential for differentiation into giant cells (*Hand1*) [34] were upregulated. These gene expression patterns indicate that the early SCNT extraembryonic compartments might have a defect in maintaining their undifferentiated status. It would be interesting to determine the upstream mechanisms that govern this SCNT-specific placental dysfunction at early postimplantation stages.

In contrast to the poor placentation at earlier stages, cloned placentas at term are typically two or three times larger than normal, primarily because of extraordinarily enlarged spongiotrophoblast and glycogen cell layers [37], [38]. Attempts have been made to identify the genes or gene networks involved in the development of placentomegaly by analyzing the global gene expression profiles of enlarged placentas with different etiologies such as those produced by SCNT, by interspecies mating and by using *Esx1* mutants [39], [40]. However, no strong candidate genes were found to be commonly dysregulated among these abnormal placentas. It is possible that multiple genes with different degrees of downregulation might concomitantly cause placental hyperplasia following SCNT, and this would hamper the identification of responsible genes by simple microarray analysis.

Our statistical analysis for DEGs common to three SCNT groups identified *Dlk1* as one of the upregulated genes in the embryonic tissue of the SCNT-derived conceptuses. This finding was confirmed by our qPCR analysis (Figure S4 in File S1). *Dlk1* is one of the imprinted genes in the *Dlk1*–*Dio3* cluster on 12qF1. This cluster consists of at least three paternally expressed protein-coding genes (*Dlk1*, *Rtl1* and *Dio3*) and four maternally expressed noncoding RNAs (*Gtl2*, *Anti-Rtl1*, *Rian* and *Mirg*) [41]. In this study, we failed to find dysregulation of imprinted genes other than *Dlk1* in this cluster (e.g., *Gtl2* and *Dio3*). This might have been because of their relatively lower expression levels at early postimplantation stages [42]. It is interesting to note that the expression pattern of these imprinted genes might serve as a marker of mouse iPS cells with the full potential to generating offspring [43], [44]. This finding suggests that this imprinted gene cluster is prone to aberrant genomic reprogramming and might critically compromise the development of the embryonic proper in both iPS-derived and SCNT-derived embryos. Consistent with this, we have observed that the placenta-only conceptuses found at term in SCNT experiments are frequently associated with loss of imprinting of the *Dlk1*–*Dio3* cluster and aberrant expression of imprinted genes in this cluster including *Dlk1* and *Gtl2* (unpublished data). Indeed, overexpression of *Dlk1* is known to have deleterious effects on embryonic development [45], although in one case, mice were viable even when expressing a bacterial artificial chromosome transgene encompassing *Dlk1* [46]. This imprinted cluster region may be the next target for the improvement of efficiency in SCNT because treatment with ascorbic acid (vitamin C) prevented loss of imprinting in this region in mouse iPS cells [47]. By contrast, many of the other common DEGs did not seem to be related to developmental processes. These genes might have been sorted from about 30,000 genes analyzed as a result of random (stochastic) variations in gene expression.

The present study was undertaken under defined genetic conditions in terms of the genotypes of the donor cells (B6D2F1), the recipient oocytes for nuclear transfer (B6D2F1) and the recipient females for embryo transfer (ICR). Such availability of strains with defined genetic backgrounds is one of the biggest advantages of the use of laboratory mice as models, although it does not always ensure that results can be extrapolated to mammals in general. The set of strains we used has been recognized to be standard in mouse SCNT experiments since the first birth of cloned mice in 1998 [23]. It is known that there is an apparent strain dependency in the efficiency of mouse cloning by SCNT, probably because of differences in the reprogrammability (or plasticity) of the genomes. The presence of the 129 strain genome in the donors has significantly increased the birth rates of clones from different cell types so far examined [4], [48]. Interestingly, this is the only inbred strain that supports full-term embryonic development at a practical level and develops cloned placentas of the normal size and structure [48], [49]. It will be interesting to identify the sets of genes responsible for the SCNT-associated phenotype – low birth rates and placental abnormalities – by comparing the gene expression profiles of early postimplantation SCNT-derived embryos.

## Supporting Information

File S1 **File containing supporting figures S1–S4.** Figure S1. Comparative global gene expression analysis between the two SCNT methods. Principle component analysis (PCA) clearly shows that global gene expression profiles of cumulus cell-derived clones generated by cell fusion (purple) or nuclear injection (red) were not distinguishable. Dark- and light-gray circles represent male and female IVF-derived control embryos, respectively. Figure S2: Expression of epiblast and extraembryonic markers (continued from Figure 1). The bar graph shows raw signal values of epiblast maker genes (*Nanog*, *Fgf5* and *Nodal*) and extraembryonic marker genes (*Krt7*, *Phlda2* and *Dnmt3L*) extracted from the microarray data. *Nanog*, *Fgf5* and *Nodal* were detected significantly in embryonic tissues, but were scant in extraembryonic samples: *Krt7*, *Phlda2* and *Dnmt3L* showed the opposite pattern. Em, embryonic samples; Ex, extraembryonic samples; IVF, control samples from *in vitro* fertilized embryos; CC, cumulus cell-derived clone; FC, fibroblast-derived clone, SC: Sertoli cell-derived clone. Figure S3: PCA of global gene expression profiles with an equivalent analysis of IVF-derived embryos (continued from Figure 2). As shown in Figure 2, gene expression similarities of embryonic samples (red, blue and green) are clearly distinguishable according to the donor cell type, while clusters of extraembryonic samples (pink, light blue and light green) overlap each other. Red, blue and green represent the embryonic samples of cumulus cell-derived clone (CC), fibroblast-derived clone (FC) and neonatal Sertoli cell-derived clone (SC) samples, respectively. Pink, light blue and light green represent the extraembryonic samples of CC, FC and SC samples, respectively. Figure S4: Quantitative reverse transcription polymerase chain reaction (qPCR) for genes in the *Dlk1–Dio3* imprint region. Gene expression levels between cumulus cell-derived clones (CC) and IVF-derived control samples were compared by qPCR. In a similar fashion to Figure 5, *Dlk1* gene expression was upregulated in embryonic samples of CC (*P* = 0.018 by one-sided Student's *t* test), though not in extraembryonic samples. There were no significant differences between the CC and IVF-derived control samples in the *Gtl2*, *Dio3* and *Rian* genes. Each dot represents the expression level relative to the *Gapdh* gene. Bars represent the means of samples in the same group.
(PDF)

Table S1 **Primer list for qPCR.**
(XLSX)

Table S2 **Overlapping DEGs among the three SCNT groups.**
(XLS)
