# Supplementary material for: Somatic Donor Cell Type Correlates with Embryonic, but Not Extra-Embryonic, Gene Expression in Postimplantation Cloned Embryos
Source: PLoS One. 2013 Oct 16;8(10):e76422. doi: 10.1371/journal.pone.0076422 (PMC3797840; doi:10.1371/journal.pone.0076422)
Supplement: File S1 — File containing supporting figures S1–S4. Figure S1. Comparative global gene expression analysis between the two SCNT methods. Principle component analysis (PCA) clearly shows that global gene expression profiles of cumulus cell-derived clones generated by cell fusion (purple) or nuclear injection (red) were not distinguishable. Dark- and light-gray circles represent male and female IVF-derived control embryos, respectively. Figure S2: Expression of epiblast and extraembryonic markers (continued from Figure 1). The bar graph shows raw signal values of epiblast maker genes (Nanog, Fgf5 and Nodal) and extraembryonic marker genes (Krt7, Phlda2 and Dnmt3L) extracted from the microarray data. Nanog, Fgf5 and Nodal were detected significantly in embryonic tissues, but were scant in extraembryonic samples: Krt7, Phlda2 and Dnmt3L showed the opposite pattern. Em, embryonic samples; Ex, extraembryonic samples; IVF, control samples from in vitro fertilized embryos; CC, cumulus cell-derived clone; FC, fibroblast-derived clone, SC: Sertoli cell-derived clone. Figure S3: PCA of global gene expression profiles with an equivalent analysis of IVF-derived embryos (continued from Figure 2). As shown in Figure 2, gene expression similarities of embryonic samples (red, blue and green) are clearly distinguishable according to the donor cell type, while clusters of extraembryonic samples (pink, light blue and light green) overlap each other. Red, blue and green represent the embryonic samples of cumulus cell-derived clone (CC), fibroblast-derived clone (FC) and neonatal Sertoli cell-derived clone (SC) samples, respectively. Pink, light blue and light green represent the extraembryonic samples of CC, FC and SC samples, respectively. Figure S4: Quantitative reverse transcription polymerase chain reaction (qPCR) for genes in the Dlk1–Dio3 imprint region. Gene expression levels between cumulus cell-derived clones (CC) and IVF-derived control samples were compared by qPCR. In a similar fashio [file pone.0076422.s001.pdf]

Figure S1

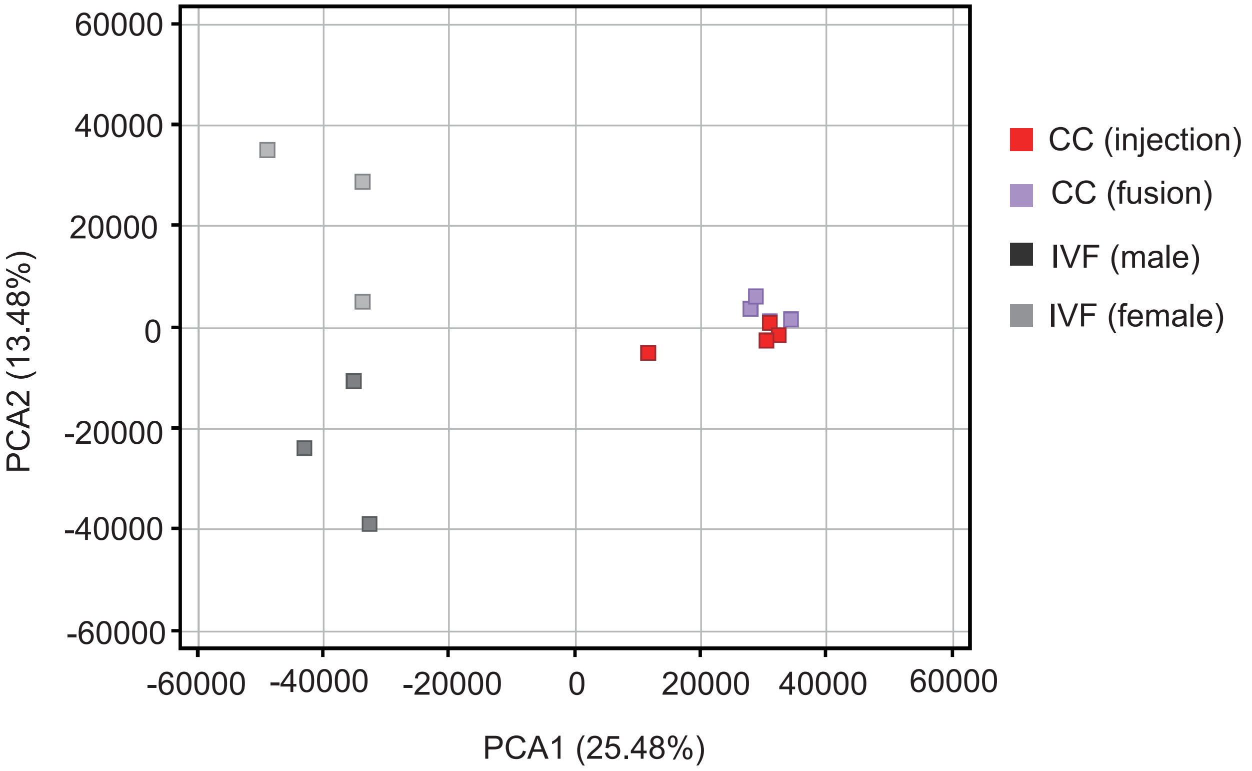

Figure S1. Comparative global gene expression analysis between the two SCNT methods. Principle component analysis (PCA) clearly shows that global gene expression profiles of cumulus cell-derived clones generated by cell fusion (purple) or nuclear injection (red) were not distinguishable. Dark- and light-gray represent male and female IVF-derived control embryos, respectively.

Figure S2

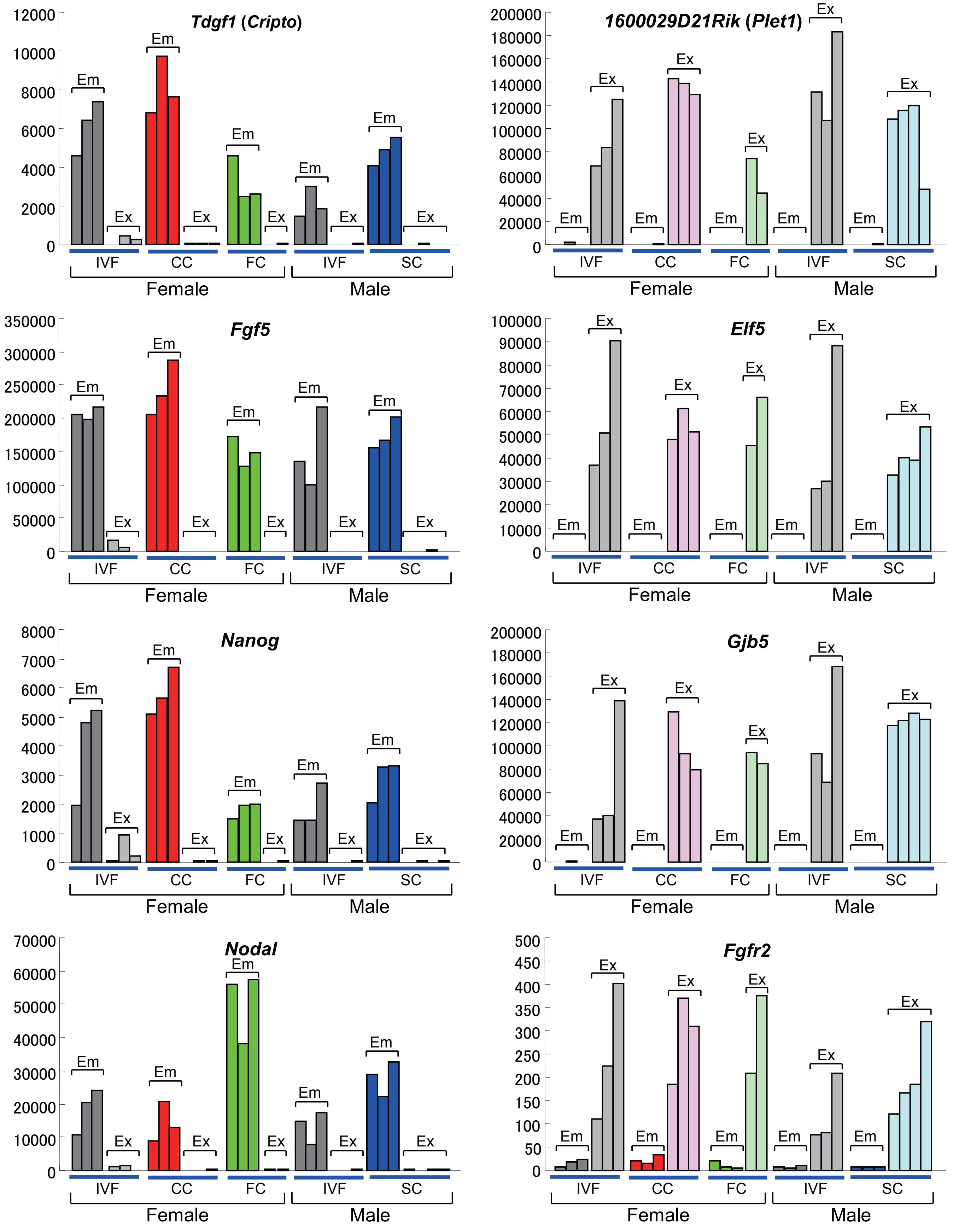

Figure S2. Expression of epiblast and extraembryonic markers (continued from Figure 1). The bar graph shows raw signal values of epiblast maker genes (*Nanog*, *Fgf5* and *Nodal*) and extraembryonic marker genes (*Krt7*, *Phlda2* and *Dnmt3L*) extracted from the microarray data. *Nanog*, *Fgf5* and *Nodal* were detected significantly in embryonic tissues, but were scant in extraembryonic samples: *Krt7*, *Phlda2* and *Dnmt3L* showed the opposite pattern. Em, embryonic samples; Ex, extraembryonic samples; IVF, control samples from *in vitro* fertilized embryos; CC, cumulus cell-derived clone; FC, fibroblast-derived clone, SC: Sertoli cell-derived clone.

Figure S3

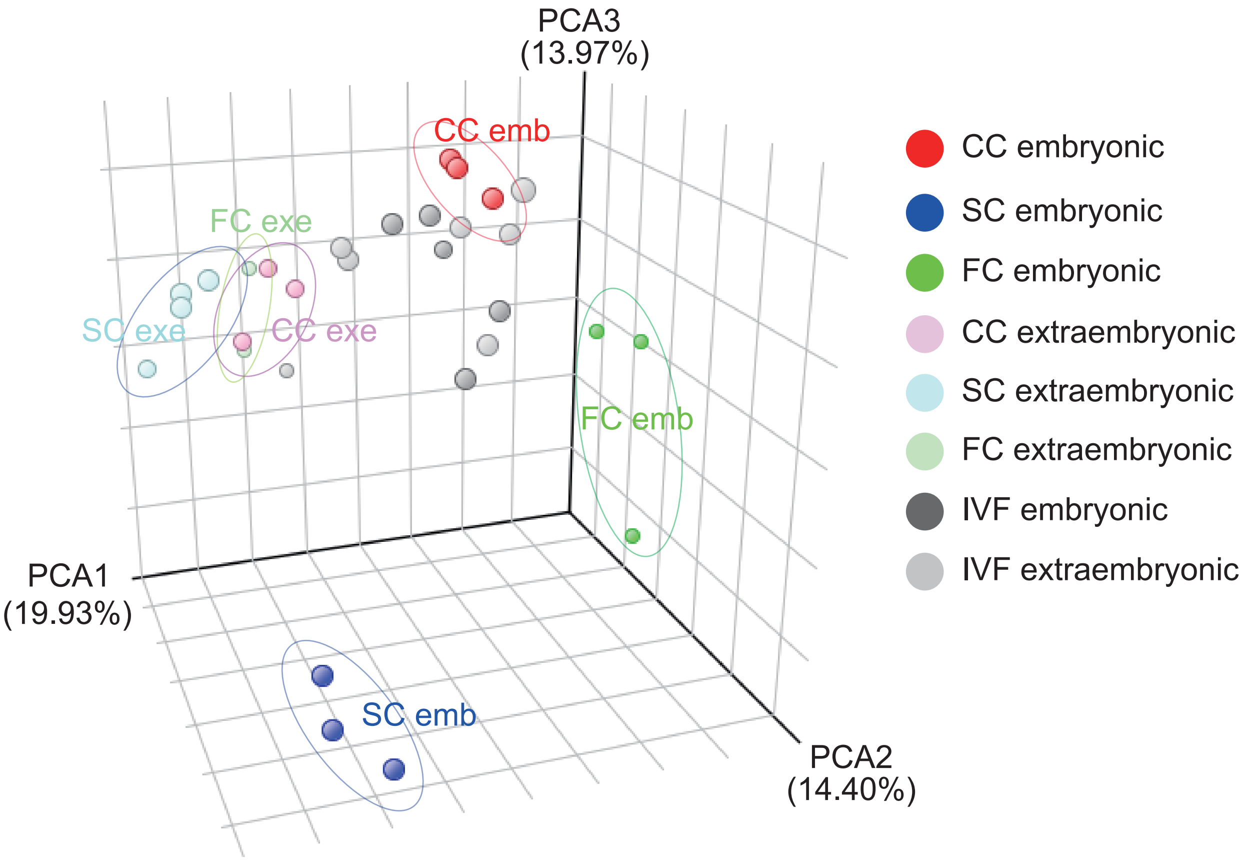

Figure S3. PCA of global gene expression profiles with an equivalent analysis of IVF-derived embryos (continued from Figure 2). As shown in Figure 2, gene expression similarities of embryonic samples (red, blue and green) are clearly distinguishable according to the donor cell type, while clusters of extraembryonic samples (pink, light blue and light green) overlap each other. Red, blue and green represent the embryonic samples of cumulus cell-derived clone (CC), fibroblast-derived clone (FC) and neonatal Sertoli cell-derived clone (SC) samples, respectively. Pink, light blue and light green represent the extraembryonic samples of CC, FC and SC samples, respectively.

Figure S4

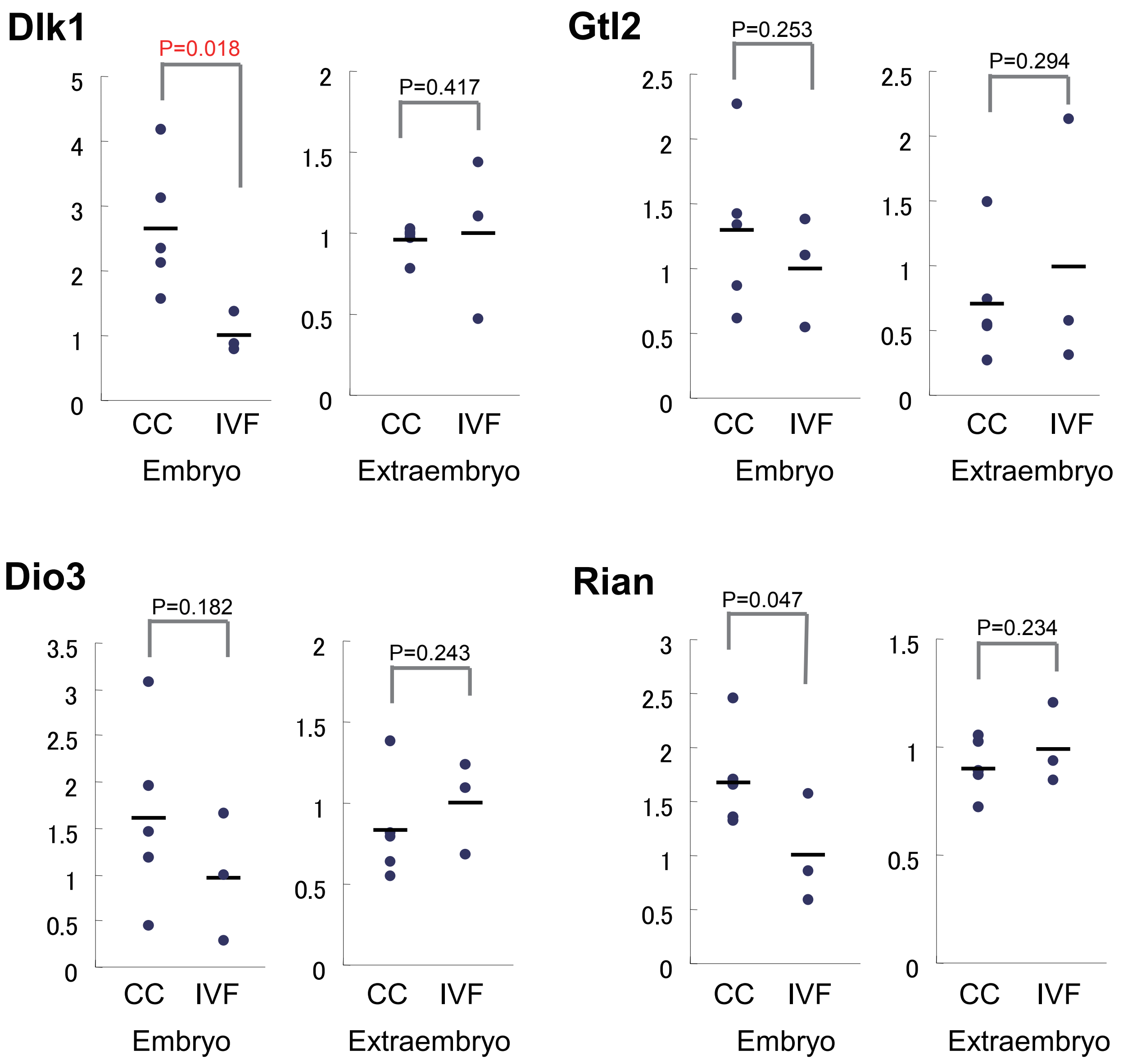

Figure S4. Quantitative reverse transcription polymerase chain reaction (qPCR) for genes in the *Dlk1–Dio3* imprint region. Gene expression levels between cumulus cell-derived clones (CC) and IVF-derived control samples were compared by qPCR. In a similar fashion to Figure 5, *Dlk1* gene expression was upregulated in embryonic samples of CC ($P = 0.018$ by one-sided Student's t test), though not in extraembryonic samples. There were no significant differences between the CC and IVF-derived control samples in the *Gtl2*, *Dio3* and *Rian* genes. Each dot represents the expression level relative to the *Gapdh* gene. Bars represent the means of samples in the same group.
